# Hsa_circ_0063804 enhances ovarian cancer cells proliferation and resistance to cisplatin by targeting miR-1276/CLU axis

**DOI:** 10.18632/aging.203474

**Published:** 2022-06-10

**Authors:** Jun You, Yuwen Han, Haifeng Qiao, Yun Han, Xiaoyan Lu, Yiling Lu, Xiaoyu Wang, Haili Kai, Yanli Zheng

**Affiliations:** 1 Department of Obstetrics and Gynecology, The Second Affiliated Hospital of Nantong University, Nantong 226001, China

**Keywords:** OC, circ_0063804, miR-1276/CLU, proliferation, drug resistance

## Abstract

Purpose: This article researched circ_0063804 effects on ovarian cancer (OC) development and resistance to cisplatin, aiming to provide a new target for OC therapy.

Methods: A total of 108 OC patients participated in this study. The circle structure of circ_0063804 was investigated using RNase R. Circ_0063804 expression in OC cells were up-regulated or down-regulated by transfection. Cell proliferation was assessed by cell counting kit-8 assay and colony formation assay. Flow cytometry was used to detect apoptosis. OC cells resistance to cisplatin was explored through MTT assay. Luciferase reporter assay was performed. qRT-PCR and Western blot was applied to research genes expression. Xenograft tumor experiment was conducted using nude mice. Ki67 expression in xenograft tumor was detected by immunohistochemistry.

Results: Circ_0063804 expression was up-regulated in OC patients and indicated poor prognosis (*P* < 0.05). Circ_0063804 had a stable circle structure. Circ_0063804 enhanced proliferation, resistance to cisplatin and reduced apoptosis of OC cells (*P* < 0.01). miR-1276 was down-regulated in OC patients and sponged by circ_0063804. CLU was directly inhibited by miR-1276 and up-regulated in OC patients. Circ_0063804 exacerbated malignant phenotype and resistance to cisplatin of OC cells *in vitro* by enhancing CLU expression via sponging miR-1276 (*P* < 0.01). Circ_0063804 silencing inhibited OC cells growth, resistance to cisplatin and Ki67 expression *in vivo* (*P* < 0.01).

Conclusion: Circ_0063804 promoted OC cells proliferation and resistance to cisplatin by enhancing CLU expression via sponging miR-1276.

## INTRODUCTION

The mortality rate of ovarian cancer (OC) is the first among gynecological malignancies. During 2018, more than 295,000 new OC patients were diagnosed and about 184,000 OC cases died [1]. However, OC cases are with a 5-year survival rate about 30%-50% [2]. Clinically, surgical resection combined with platinum compound chemotherapy is the traditional treatment method for OC. However, more than 70% OC patients eventually suffered recurrence and metastasis [3]. The main reason for the high mortality and recurrence rate of OC is the lack of high-sensitivity and high-specificity of early diagnosis technology and long-term effective treatment options. Therefore, the search for effective therapeutic targets has become a focus in OC treatment field in recent years.

Non-coding RNAs is a type of RNA transcript that does not have protein-coding potential, including microRNAs (miRNAs), long chain non-coding RNAs (lncRNAs) and circular RNAs (circRNAs). It is not only participated in regulating a series of biological processes, but also involved in tumorigenesis, metastasis and prognosis of tumor patients [4]. CircRNAs is a new endogenous non-coding RNA that has only been discovered in recent years. It is abundant in eukaryotic cells with a large variation in length ranged from hundreds to thousands nucleotides [5, 6]. In comparison with linear RNA forms, circRNAs is covalently closed RNA molecules, which makes it more stable in structure [7]. The most well-known mechanism for circRNAs to participate in pathological development is to regulate mRNAs expression via acting as competing endogenous miRNAs sponge elements [8]. Recently, circRNAs is increasingly found to exert a vital role in the tumorigenesis and development of malignant tumors.

Circ_0063804 is a novel circRNA, which function in OC and other human malignancies has never been revealed. Thus, this article investigated the effect of circ_0063804 on OC development and cisplatin resistance. In our preliminary research, we noticed that circ_0063804 had binding site for miR-1276 and clusterin (CLU) possessed binding site for miR-1276. Then this paper speculated that circ_0063804 might regulate OC development and cisplatin resistance through regulating the miR-1276/CLU axis. This study verified this speculation by performing a series of experiments. We hope that this paper would provide potential novel target for OC clinical therapy.

## MATERIALS AND METHODS

### Patients and clinical tissues

In this research, 108 pairs of tumor tissues and corresponding adjacent normal tissues were obtained from OC cases. All cases were initially identified as OC from October 2013 to April 2015. Clinical tissue samples were immediately put into in liquid nitrogen for storage after obtained. The clinical features of patients were recorded, including age, FIGO stage, distant metastasis, tumor grade and size (exhibited in Table 1). After surgery, patient's follow-up time was 60 months.

**Table 1:** Relationship between circ_0063804 expression and clinical features of OC patients.

**Characteristics**	**Number of patients**	**circ_0063804 Low expression (< median)**	**circ_0063804 High expression (≥ median)**	***P* value**
Number	108	52	56	
Ages (years)				0.497
< 55	53	25	28	
≥ 55	55	27	28	
FIGO stage				0.018
I & II	50	30	20	
III & IV	58	22	36	
Grade				0.029
G1	49	29	20	
G2 & G3	59	23	36	
Distant metastasis				0.508
Yes	51	25	26	
No	57	27	30	
Tumor size (cm)				0.006
≤ 10	48	30	18	
> 10	60	22	38	

FIGO: International Federation of Gynecology and Obstetrics. OC: ovarian cancer.

This study has obtained the written informed consent from all participants, and has approved by the Ethics Committee of The Second Affiliated Hospital of Nantong University. Researches involved in this paper were carried out in compliance with the Helsinki Declaration.

### Cell culture

Human ovarian epithelial cell line (HOSEPiC) and 7 human OC cell lines (SKOV3, OV420, A2780, HO8910, CAOV3, OVCAR4, OVCAR3), were commercially obtained from the Institute of Biochemistry and Cell Biology of the Chinese Academy of Sciences (Shanghai, China). Cell lines were separately grown in Dulbecco’s Modified Eagle Medium (DMEM) containing 10% fetal bovine serum (FBS) at 37° C, 5% CO_2_.

### RNase R digestion

The circle structure of circ_0063804 was investigated by incubating with RNase R (Solarbio, Beijing, China). Briefly, in line with the instructions, total RNA was extracted from OC clinical tissue samples using Trizol reagent (Solarbio, Beijing, China). Then total RNA sample (5 μg) was treated by RNase R (3 U/μg) for 20 min at 37° C. Meanwhile, equal amount of total RNA sample was kept at 37° C for 20 min, but without the addition of RNase R. The linear RNA corresponding to circ_0087862 was CELSR1. The expression of circ_0087862 and liner RNA CELSR1 was explored by quantitative real time polymerase chain reaction (qRT-PCR).

### Transfection

The short hairpin RNA (shRNA: 3'-GAGCTGTTATAATCGAGAGCA-5') targeting circ_0063804 and negative control (shNC) were provided by GenePharma (Shanghai, China). The full-length sequences of circ_0063804 and CLU (GenePharma, Shanghai, China) were separately cloned into pCDNA 3.1 vector (Mingjing Biology, Shanghai, China) based on the manuals. OVCAR3 cells were grown in serum-free DMEM, and then transfected by circ_0063804 shRNA and shNC respectively (named sh-circ_0063804 group and sh-NC group). SKOV3 cells grown in DMEM (without FBS) were separately subjected to transfection by pCDNA 3.1-circ_0063804 vectors and empty pCDNA 3.1 vectors (named oe-circ_0063804 group and oe-NC group). miR-1276 mimics, mimics control, miR-1276 inhibitor and inhibitor control were all provided by GenePharma (Shanghai, China). miR-1276 mimics and corresponding mimics control were respectively transfected into OVCAR3 cells (named miR-1276 mimic group and miR-NC group). miR-1276 inhibitor and inhibitor control were separately transfected into SKOV3 cells (named miR-1276 inhibitor group and inh-NC group). Cotransfection was performed on OVCAR3 cells using both circ_0063804 shRNA and miR-1276 inhibitor (named sh-circ_0063804 + miR-1276 inhibitor group) or both circ_0063804 shRNA and pCDNA 3.1-CLU (named sh-circ_0063804 + oeCLU group). Lipofectamine 2000 (Thermo Fisher Scientific, Waltham, MA, USA) was applied for transfection in line with the instructions. Eight hour post-transfection, cells were maintained in DMEM (10% FBS) for 48 hour at 37° C, 5% CO_2_. Cells transfection efficiency was explored by qRT-PCR.

### Cell counting kit-8 (CCK-8) assay

Cells were harvested at 48 hour post-transfection, and then suspended in DMEM (with 10% FBS) (1 × 10^5^ cells/mL). Using sterile pipettes, the cell suspension (100 μL) was grown into 96-well plates. Plates were kept at 37° C, 5% CO_2_. At 12, 24, 48 and 72 hour, CCK-8 reagent (10 μL) was used to treat cells for 4 hour at 37° C. A microplate reader (Biotek, USA) was applied for optical density (OD) value detection at 450 nm.

### Colony formation assay

Cells were collected at 48 hour post-transfection. DMEM (10% FBS) was used to suspend cells (500 cells/mL). The cell suspension (1 mL) was grown into 6-well plates. Cells were kept at 37° C, 5% CO_2_. DMEM (10% FBS) in each well was replaced every three days. After two weeks, the residual liquid in each well was removed. Paraformaldehyde (4%) was applied to treat cells for 20 min. Crystal violet (0.1%) was then responsible for 10 min staining of cells. Under a microscope (Olympus, Tokyo, Japan), cell colonies with more than 50 cells were counted.

### Flow cytometry

At 48 hour post-transfection, cells were digested by trypsin and washed by phosphate buffered saline (PBS) for 3 times. At 1000 rpm, cells were centrifuged for 5 min. Then 1 × 10^5^ cells were dispersed in 1 × Binding Buffer (500 μL) in tubes. Annexin V-fluorescein isothiocyanate (Annexin V-FITC) (5 μL) and propidium iodide (PI) (5 μL) was applied to treat cells. Cells were then kept for 15 min at room temperature in the dark. The apoptosis was determined by flow cytometry.

### Cisplatin resistance test

The cisplatin resistance of OVCAR3 and SKOV3 cells was explored through 3-(4, 5-Dimethylthiazol-2-yl)-2, 5-diphenyltetrazolium bromide (MTT) assay. In short, cells were grown in 96-well plates (5000 cells/well) overnight with DMEM (10% FBS). Cisplatin with varying final concentration (1000, 250, 62.5, 15.625, 3.9, 0.97 and 0.24 nM) was then added to treat cells. Cells were maintained at 37° C, 5% CO_2_ for 48 hour. After that, MTT solution (5 mg/mL, 20 μL) was dripped into wells. Cells were kept at 37° C for 4 hour. After the residual liquid in wells being discarded, dimethyl sulfoxide (DMSO, 150 μL) was then added into wells. After the crystal was completely dissolved, the OD value of wells was explored by a microplate reader at 450 nm. The cisplatin dose that suppressed cell growth by 50% (IC50) was calculated through LOGIT method.

### Cisplatin and DMSO treatment

Cisplatin or DMSO was dispersed in DMEM (10% FBS) to a final dose of 100 nM. OVCAR3 and SKOV3 cells without any treatment were grown in 6-well plates(1 × 10^5^ cells/well). Afterwards, DMEM (10% FBS) and cisplatin (or DMSO) was added into each well with a volume of 1 mL [9]. Cells were used as Cisplatin group or DMSO group. In addition, the transfected OVCAR3 and SKOV3 cells were also inoculated into 6-well plates, and incubated by DMEM (10% FBS) and cisplatin (or DMSO). These cells were named DMSO + sh-circ_0063804 group, Cisplatin + sh-circ_0063804 group, DMSO + oe-circ_0063804 group and Cisplatin + oe-circ_0063804 group respectively. After 48 hour of incubation at 37° C, 5% CO_2_, the expression of p-gp, PARP, c-PARP proteins expression in cells of each group was investigated by Western blot.

### Luciferase reporter assay

Circular RNA Interactome and miRDB was used for the prediction of target genes for circ_0063804. TargetScan was applied for the prediction of target genes for miR-1276. Online prediction revealed that miR-1276 contained binding site for circ_0063804 and CLU contained the binding site for miR-1276. Luciferase reporter assay was then applied to research the interaction between two genes. According to the binding site, circ_0063804-wildtype (WT) fragment, circ_0063804-mutant type (Mut) fragment, CLU-WT fragment and CLU-Mut fragment, were commercially provided by GenePharma (Shanghai, China). According to the instructions, the 4 kinds of fragments were separately loaded onto the luciferase reporter vectors. OVCAR3 cells were transfected by miR-1276 mimics (served as miR-1276 mimic group) and mimics control respectively (used as miR-NC group). These cells were then cotransfected by the luciferase reporter vectors containing the four type fragments. Lipofectamine 2000 (Thermo Fisher Scientific, Waltham, MA, USA) was responsible for transfection. Cells were maintained for 48 hour at 37° C, 5% CO_2_. Afterwards, the luciferase activity was investigated by the Double-Luciferase Reporter assay system (Promega, Madison, WI, USA). By normalizing to Renilla luciferase activity, the relative firefly luciferase activity was calculated.

### qRT-PCR

Total RNA was obtained from cells/tissues through using Trizol reagent (Solarbio, Beijing, China). Reverse transcription was completed with 1 μg of each total RNA sample using the PrimeScript RT Reagent Kit (Takara, Dalian, China). Based on the instructions, real time-PCR was carried out by the SYBR Premix Ex Taq (Takara, Otsu, Shiga, Japan). The reaction procedure was 95° C for 10 min at first, and then 40 cycles of 95° C for 15 s and 60° C for 60 s. U6 and GAPDH was served as the control. Genes relative expression was determined with 2^-ΔΔCt^ method. Specific primers used were as follows: circ_0063804, forward 5’-TCGCTTCAAGACACCCTGATTT-3’ and reverse 5’-CAGTGGAAGCCGCCGATGA-3’. miR-1276, forward 5'-TAGGTAAAGAGCCCTGTGGAGA-3' and reverse 5'-CATCAAGGCCCAAGTGCTCAG-3'. U6, forward 5'-CTCGCTTCGGCAGCACA-3' and reverse 5'-ACGCTTCACGAATTTGC-3'. CLU, forward 5'-ACGCGTCGACACATGTCCAATCAGGGAAGTAAG-3' and reverse 5'-TTCGCCGGCGTCTCACTCCTCCCGGTGCTTTTT-3'. GAPDH, forward 5'-CTCTGCTCCTCCTGTTCGAC-3' and reverse 5'-ACCAAATCCGTTGACTCCGA-3'.

### Western blot

The whole extracts from OVCAR3 and SKOV3 cells were prepared using RIPA lysis buffer (Promega, Madison, WI, USA). The total proteins concentration in the extracts was quantified by the BCA kit (Beyotime, Shanghai, China). The same amount of each total proteins sample was underwent 12% sodium dodecyl sulphate polyacrylamide gel electrophoresis (SDS-PAGE). Proteins were treated with 5% skimmed milk after transferred to polyvinylidene fluoride (PVDF) membranes. In a 4° C refrigerator, the membranes were probed by primary antibodies (1:1000 dilution) for 12 hour. Rabbit anti-PARP, rabbit anti-c-PARP, and rabbit anti-CLU was provided by Santa Cruz (Dallas, TX, USA). Mouse anti-p-gp and rabbit anti-β-actin was commercially obtained from Cell Signaling Technology (Danvers, MA, USA). The membranes were then treated for 2 hour by horseradish peroxidase-conjugated secondary antibody (1:5000, Solarbio, Beijing, China) at room temperature. Protein blots were exposed by treating with enhanced chemiluminescence (ECL). The gray value of protein blots was quantified by Quantity One software (Bio-Rad, Hercules, CA, USA). In this article, β-actin was the control.

### Xenograft tumor experiment

A total of 24 nude mice (4-5 weeks old) were kept in a room at 25° C with free access to water and food. All mice were commercially provided by Shanghai Experimental Animal Center of Chinese Academy of Sciences (Shanghai, China). Animal experiments in this paper have been approved by the ethics committee of The Second Affiliated Hospital of Nantong University. The animal experiments were in line with the Guide for the Care and Use of Laboratory Animals.

Mice were set into 4 groups: sh-NC group (n = 6), sh-NC + cisplatin group (n = 6), sh-circ_0063804 group (n = 6) and sh-circ_0063804 + cisplatin group (n = 6). OVCAR3 cells transfected by circ_0063804 shNC were injected subcutaneously into the dorsal flanks of mice in sh-NC group and sh-NC + cisplatin group. At the same injection site, OVCAR3 cells transfected by circ_0063804 shRNA were injected into mice in sh-circ_0063804 group and sh-circ_0063804 + cisplatin group. The number of injected cells was 5 × 10^6^ cells. Notably, for mice of sh-NC + cisplatin group and sh-circ_0063804 + cisplatin group, they were given an intraperitoneal injection of cisplatin (5 mg/kg) every day. Cisplatin was dissolved in a sterile solution containing NaCl, citric acid and water. The size of xenograft tumor was monitored every 7 days by the formula of 0.5 × tumor width^2^ × length. On the 28^th^ day post-injection, mice were killed through rapid neck dislocation. Subcutaneous xenograft tumor was then obtained and weighted.

### Immunohistochemistry

Xenograft tumors were fixed in 10% formaldehyde. Paraffin was then applied to embed xenograft tumors, and the xenograft tumors were then prepared into sections (4 μm in thickness). After being dewaxed by xylene and rehydrated by gradient alcohol, 0.01 M citrate buffer was applied to treat sections. Blocking of endogenous peroxidase was performed by 15 min incubation with H_2_O_2_ solution (3%). Goat serum (5%) was applied to treat sections for half an hour. Mouse anti-human Ki67 monoclonal antibody (1:100, Cell Signaling Technology, Danvers, MA, USA) was selected to probe sections for 12 hour at 4° C. At room temperature, horseradish peroxidase labeled secondary antibody (1:200, Boster, Wuhan, China) was selected to perform 30 min incubation of sections. Three times washing by PBS were carried out on sections. The sections were then subjected to staining through diaminobenzidine (DAB) solution and hematoxylin solution. Ki67 positive expression cells (brown) were visualized under a microscope (Olympus, Tokyo, Japan).

### Statistical analysis

Experiments involved in this article were independently carried out for 3 times. Data were shown as mean ± standard deviation (SD) and processed by SPSS19.0 software (SPSS Inc., Chicago, IL, USA). In this research, difference comparison between two groups was conducted with two-tailed paired Student's t-test. Differences in more than two groups were compared by one-way analysis of variance (ANOVA) with Tukey's post hoc test for pairwise comparisons. Kaplan-Meier curve was made to research OC cases survival. The correlation between circ_0063804 expression and clinical features of OC cases was assessed via Pearson's χ2 test. Spearman's correlation analysis was selected to explore the correlation between two genes expression in OC tissues. *P* < 0.05 revealed a significant difference.

## RESULTS

### High circ_0063804 expression in OC patients indicated poor prognosis

**Figure 1:**
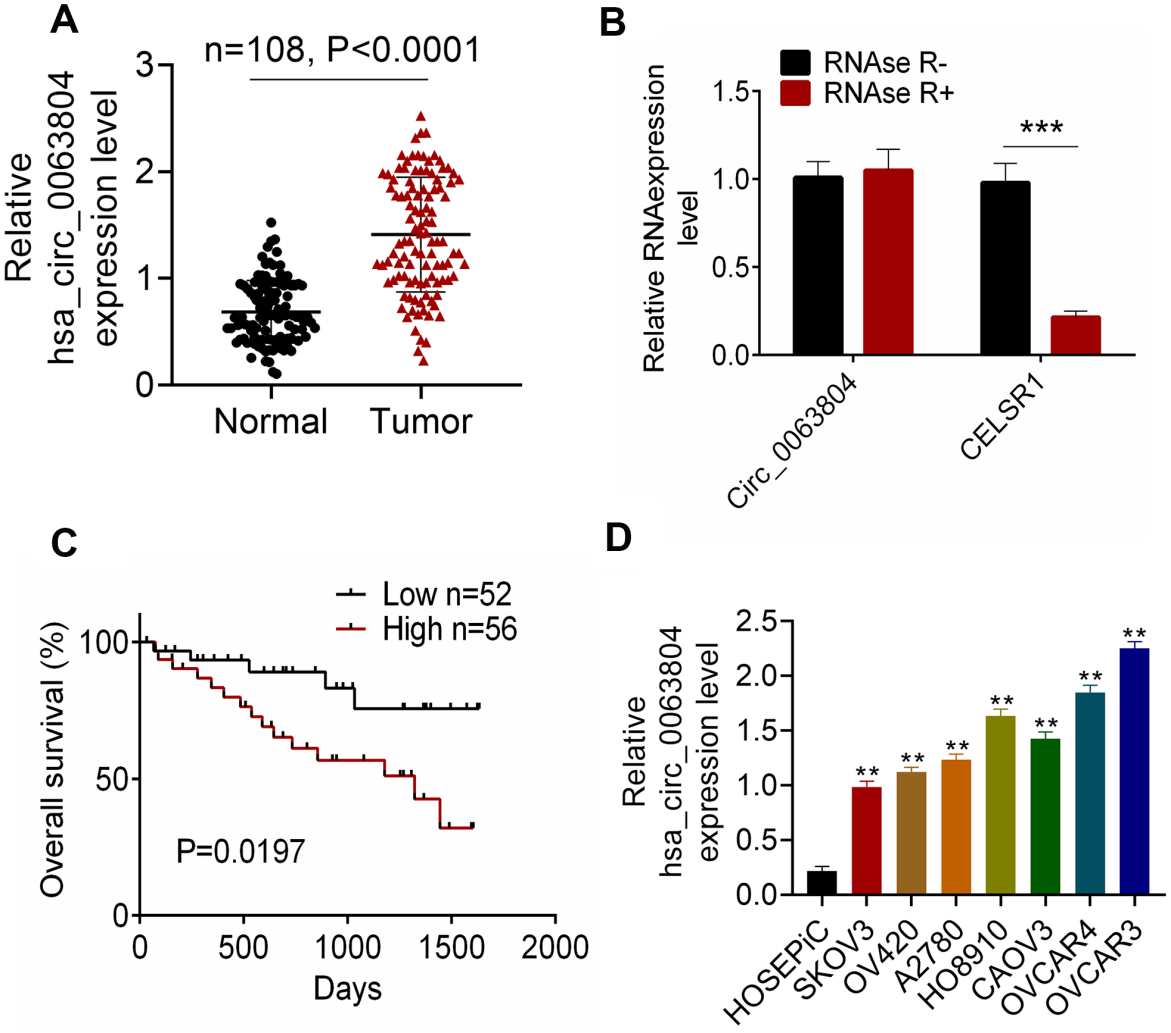
**Circ_0063804 expression in OC patients was up-regulated.** (**A**) Circ_0063804 expression in tumor tissues of OC patients was aberrantly increased than that in normal tissues. (**B**) The stable circle structure of circ_0063804 was confirmed by using RNase R. (**C**) High circ_0063804 expression indicated low survival rate of OC patients. (**D**) Circ_0063804 expression in human OC cell lines was significantly higher than that in human ovarian epithelial cell line. ** *P* < 0.01. *** *P* < 0.001. OC: ovarian cancer.

Of the 108 OC patients, circ_0063804 expression in OC tumor tissues was aberrantly increased than that in matched adjacent normal tissues (*P* < 0.0001) (Figure 1A). The circle structure of circ_0063804 was investigated using RNase R. The addition of RNase R seriously reduced the expression of linear RNA CELSR1 (*P* < 0.001) (Figure 1B). However, circ_0063804 expression did not change obviously whether RNase R was added or not. Therefore, circ_0063804 had a stable circle structure. Notably, high circ_0063804 expression was closely related to lower survival (*P* = 0.0197), advanced FIGO stage (*P* < 0.05) and grade, and larger tumor size (*P* < 0.01) (Figure 1C and Table 1). *In vitro* experiment showed that, circ_0063804 expression in human OC cell lines (SKOV3, OV420, A2780, HO8910, CAOV3, OVCAR4, OVCAR3) was significantly higher than that in human ovarian epithelial cell line (HOSEPiC) (*P* < 0.01). Of which, SKOV3 exhibited the lowest circ_0063804 expression and OVCAR3 showed the highest circ_0063804 expression (Figure 1D). Therefore, in subsequent research, OVCAR3 and SKOV3 cells were used as research objects to study circ_0063804 effects on OC development and cisplatin resistance.

### Circ_0063804 silencing promoted OC cells apoptosis and weakened proliferation and resistance to cisplatin

**Figure 2:**
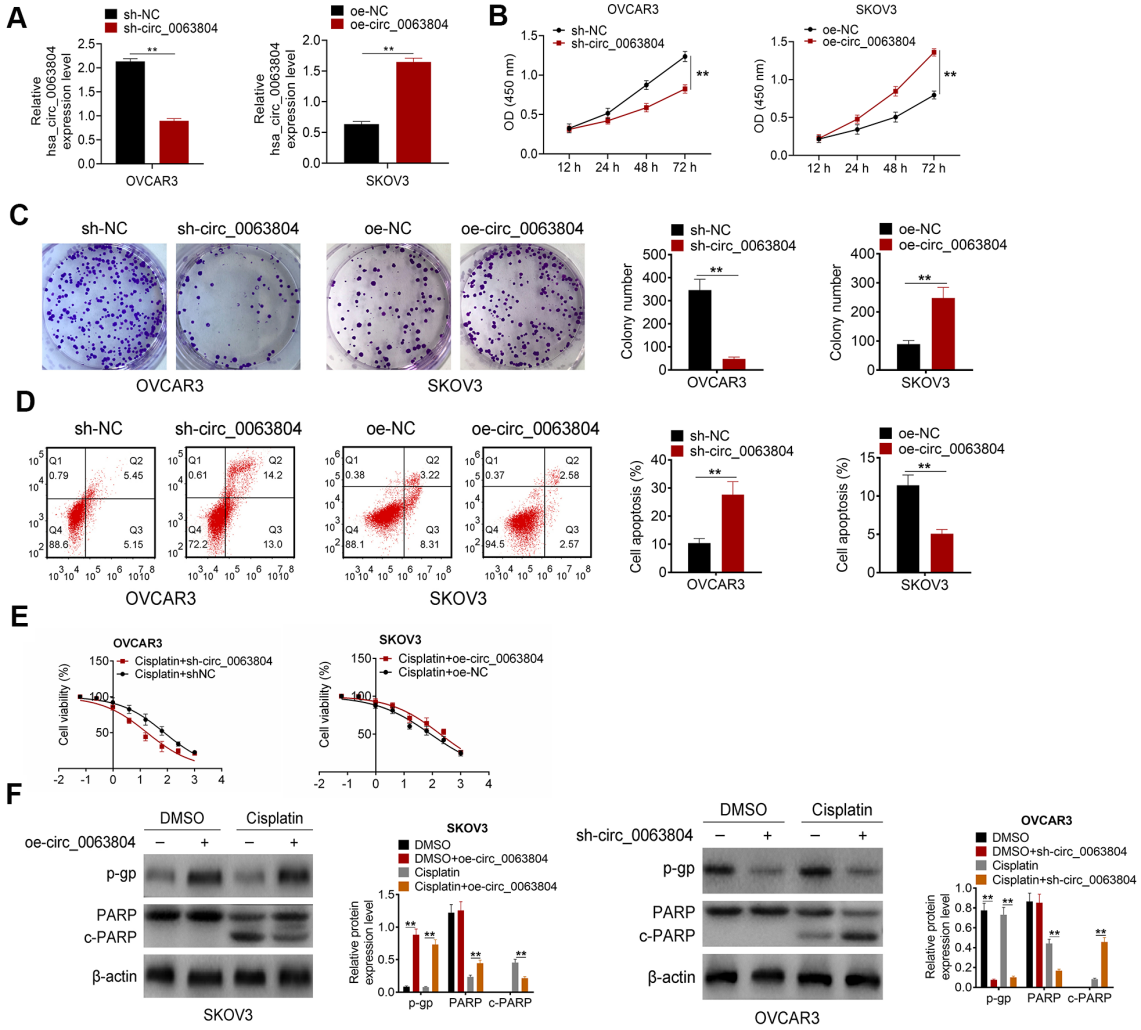
**Circ_0063804 silencing promoted OC cells apoptosis and weakened proliferation and resistance to cisplatin.** (**A**) Circ_0063804 expression in OC cells was successfully regulated via transfection. (**B**, **C**) According to CCK-8 assay and colony formation assay, circ_0063804 silencing inhibited OC cells proliferation, and circ_0063804 overexpression promoted OC cells proliferation. (**D**) Flow cytometry revealed that circ_0063804 silencing promoted OC cells apoptosis, and circ_0063804 overexpression inhibited OC cells apoptosis. (**E**) MTT assay indicated that circ_0063804 silencing reduced the IC50 of OC cells, and circ_0063804 overexpression elevated the IC50 of OC cells. (**F**) Circ_0063804 silencing reduced OC cells resistance to cisplatin, and circ_0063804 overexpression enhanced OC cells resistance to cisplatin. ** *P* < 0.01. CCK-8: cell counting kit-8. MTT: 3-(4,5-Dimethylthiazol-2-yl)-2,5-diphenyltetrazolium bromide. OC: ovarian cancer.

From Figure 2A, OVCAR3 cells of sh-circ_0063804 group expressed much declined circ_0063804 relative to sh-NC group (*P* < 0.01). SKOV3 cells of oe-circ_0063804 group displayed distinctly increased circ_0063804 expression compared to oe-NC group (*P* < 0.01). The data demonstrated that circ_0063804 expression in OVCAR3 and SKOV3 cells was effectively regulated via transfection.

CCK-8 assay and colony formation assay were both applied to research cells proliferation ability. Distinctly lower OD450 value and colony number was seen in OVCAR3 cells of sh-circ_0063804 group matched with sh-NC group (*P* < 0.01). Oppositely, matched with oe-NC group, remarkably elevated OD450 value and colony number was seen in SKOV3 cells of oe-circ_0063804 group (*P* < 0.01) (Figure 2B, 2C). From flow cytometry, OVCAR3 cells of sh-circ_0063804 group presented much higher apoptotic percentage than sh-NC group (*P* < 0.01). However, the apoptotic percentage was obviously declined in SKOV3 cells of oe-circ_0063804 group when relative to oe-NC group (*P* < 0.01) (Figure 2D). The IC50 was reflected using MTT assay. The IC50 of OVCAR3 cells in Cisplatin + sh-NC group and Cisplatin + sh-circ_0063804 group was 76.36 μg/mL and 17.64 μg/mL respectively. Meanwhile, the IC50 of SKOV3 cells in Cisplatin + oe-NC group and Cisplatin + oe-circ_0063804 group was 85.67 μg/mL and 188 [4]. μg/mL respectively (Figure 2E). Western blot displayed that, matched with DMSO group, OVCAR3 cells of DMSO + sh-circ_0063804 group were with pronounced down-regulation of p-gp protein (*P* < 0.01). Matched with Cisplatin group, OVCAR3 cells of Cisplatin + sh-circ_0063804 group showed distinctly down-regulation of p-gp, PARP proteins and up-regulation of c-PARP protein (*P* < 0.01). However, SKOV3 cells of DMSO + oe-circ_0063804 group had severely up-regulation of p-gp protein than DMSO group (*P* < 0.01). When compared to Cisplatin group, SKOV3 cells of Cisplatin + oe-circ_0063804 group were displayed dramatically up-regulation of p-gp, PARP proteins and down-regulation of c-PARP protein (*P* < 0.01) (Figure 2F).

### miR-1276 was sponged by circ_0063804 and down-regulated in OC patients

**Figure 3:**
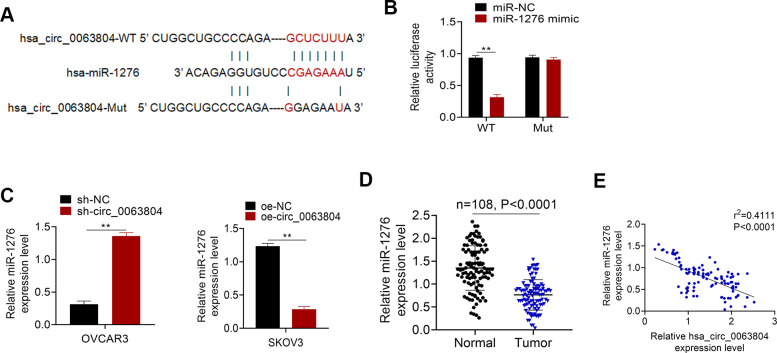
**miR-1276 was sponged by circ_0063804 and down-regulated in OC patients.** (**A**) The binding site of circ_0063804-WT or -Mut for miR-1276. (**B**) Luciferase reporter assay identified that miR-1276 was a target gene of circ_0063804. (**C**) Circ_0063804 silencing elevated miR-1276 expression, and circ_0063804 overexpression reduced miR-1276 expression in OC cells. (**D**) miR-1276 expression was down-regulated in tumor tissues of OC patients. (**E**) The expression of circ_0063804 and miR-1276 was negatively correlated in tumor tissues of OC patients. ** *P* < 0.01. OC: ovarian cancer.

Figure 3A presented the binding site of circ_0063804-WT or -Mut for miR-1276. Based on Luciferase reporter assay, OVCAR3 cells of miR-1276 mimic group had seriously lower relative circ_0063804-WT luciferase reporter luciferase activity than miR-NC group (*P* < 0.01). There was no obvious difference in the relative circ_0063804-Mut luciferase reporter luciferase activity between miR-1276 mimic group and miR-NC group (Figure 3B). Markedly higher miR-1276 expression level was seen in OVCAR3 cells of sh-circ_0063804 group when matched with sh-NC group (*P* < 0.01). Conversely, remarkably lower miR-1276 expression was shown in SKOV3 cells of oe-circ_0063804 group when relative to oe-NC group (*P* < 0.01) (Figure 3C). In OC cases, significantly reduced miR-1276 expression level was presented in clinical tumor tissues than that in matched adjacent normal tissues (*P* < 0.0001) (Figure 3D). circ_0063804 level was negatively correlated with miR-1276 level in tumor tissues of OC cases (*P* < 0.0001) (Figure 3E).

### CLU was directly inhibited by miR-1276 and overexpressed in OC cases

**Figure 4:**
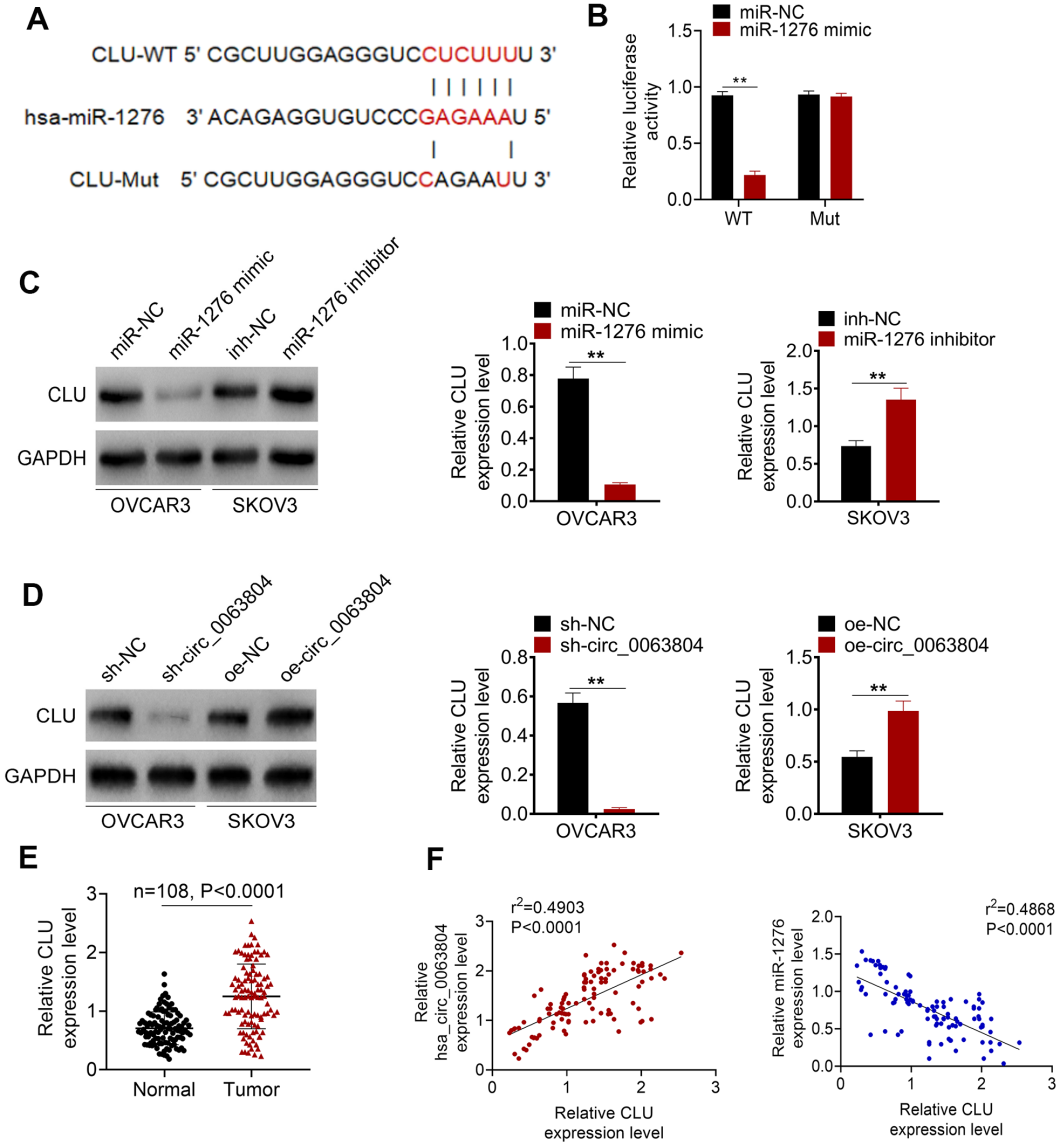
**CLU was directly inhibited by miR-1276 and up-regulated in OC patients.** (**A**) The binding site of CLU-WT or -Mut for miR-1276 was shown. (**B**) Luciferase reporter assay showed that CLU was a target gene of miR-1276. (**C**) miR-1276 up-regulation decreased CLU expression, and miR-1276 down-regulation increased CLU expression. (**D**) circ_0063804 down-regulation reduced CLU expression, and circ_0063804 up-regulation elevated CLU expression. (**E**) CLU expression was dramatically increased in tumor tissues than that in normal tissues of OC patients. (**F**) In OC tissues, CLU expression level was positively correlated with circ_0063804 expression, and negatively correlated with miR-1276 expression. ** *P* < 0.01. OC: ovarian cancer.

The binding site of CLU-WT or -Mut for miR-1276 was exhibited in Figure 4A. Luciferase reporter assay was performed using OVCAR3 cells. Compared with miR-NC group, significantly decreased relative CLU-WT luciferase reporter luciferase activity was observed in OVCAR3 cells of miR-1276 mimic group (*P* < 0.01). No obvious change was occurred in the relative CLU-Mut luciferase reporter luciferase activity between miR-NC group and miR-1276 mimic group (Figure 4B). Western blot showed that, OVCAR3 cells of miR-1276 mimic group had markedly down-regulation of CLU protein than miR-NC group (*P* < 0.01). Oppositely, SKOV3 cells of miR-1276 inhibitor group had obviously up-regulation of CLU protein than inh-NC group (*P* < 0.01) (Figure 4C). Moreover, matched with sh-NC group, markedly down-regulation of CLU protein was exhibited in OVCAR3 cells of sh-circ_0063804 group (*P* < 0.01). Conversely, matched with oe-NC group, SKOV3 cells of oe-circ_0063804 group exhibited prominently up-regulation of CLU protein (*P* < 0.01) (Figure 4D). In clinical samples of OC cases, CLU expression level was dramatically higher in tumor tissues than that in matched adjacent normal tissues (*P* < 0.0001) (Figure 4E). In OC tissues, CLU expression level was positively related to circ_0063804 expression, but negatively correlated with the expression of miR-1276 (*P* < 0.0001) (Figure 4F).

### Circ_0063804 promoted OC development *in vitro* by facilitating CLU expression through sponging miR-1276

**Figure 5:**
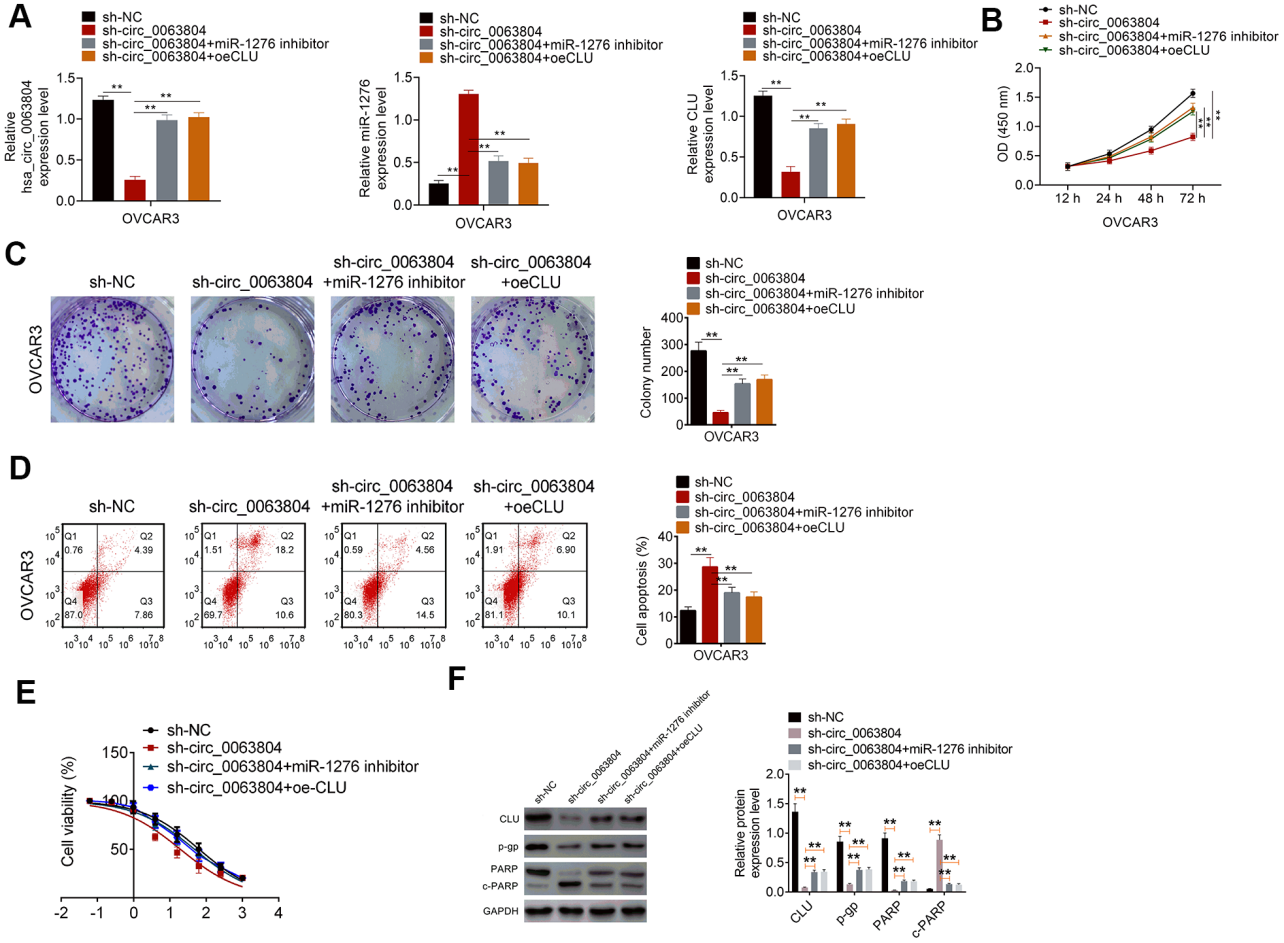
**Circ_0063804 promoted OC development *in vitro* by enhancing CLU expression via sponging miR-1276.** (**A**) circ_0063804 could enhance CLU expression via sponging miR-1276. (**B**, **C**) CCK-8 assay and colony formation assay revealed that, circ_0063804 promoted OVCAR3 cells proliferation through promoting CLU expression via sponging miR-1276. (**D**) circ_0063804 inhibited OVCAR3 cells apoptosis by enhancing CLU expression via sponging miR-1276. (**E**) circ_0063804 elevated the IC50 of OVCAR3 cells by enhancing CLU expression via sponging miR-1276. (**F**) circ_0063804 enhanced CLU, p-gp and PARP proteins expression, and weakened c-PARP protein expression through promoting CLU expression via sponging miR-1276. ** *P* < 0.01. CCK-8: cell counting kit-8. OC: ovarian cancer.

As shown in Figure 5A, OVCAR3 cells of sh-circ_0063804 group expressed much lower circ_0063804, CLU expression and higher miR-1276 expression when matched with sh-NC group (*P* < 0.01). Matched with sh-circ_0063804 group, prominently higher circ_0063804, CLU expression and lower miR-1276 expression was found in sh-circ_0063804 + miR-1276 inhibitor group and sh-circ_0063804 + oeCLU group (*P* < 0.01). These data further indicated that circ_0063804 could enhance CLU expression via sponging miR-1276.

Functional analysis presented that, compared with sh-NC group, significantly lower OD450 value, colony number and higher apoptotic percentage was occurred in OVCAR3 cells of sh-circ_0063804 group (*P* < 0.01). Matched withsh-circ_0063804 group, remarkably higher OD450 value, colony number and lower apoptotic percentage was observed in sh-circ_0063804 + miR-1276 inhibitor group and sh-circ_0063804 + oeCLU group (*P* < 0.01) (Figure 5B–5D). The IC50 of OVCAR3 cells in sh-NC group, sh-circ_0063804 group, sh-circ_0063804 + miR-1276 inhibitor group and sh-circ_0063804 + oeCLU group was 63.47 μg/mL, 19.34 μg/mL, 47.32 μg/mL and 52.74 μg/mL, respectively (Figure 5E). Relative to sh-circ_0063804 group, OVCAR3 cells of sh-NC group, sh-circ_0063804 + miR-1276 inhibitor group and sh-circ_0063804 + oeCLU group exhibited prominently up-regulation of CLU, p-gp and PARP proteins, and down-regulation of c-PARP protein (*P* < 0.01) (Figure 5F).

### Circ_0063804 silencing weakened OC cells *in vivo* growth and cisplatin resistance

**Figure 6:**
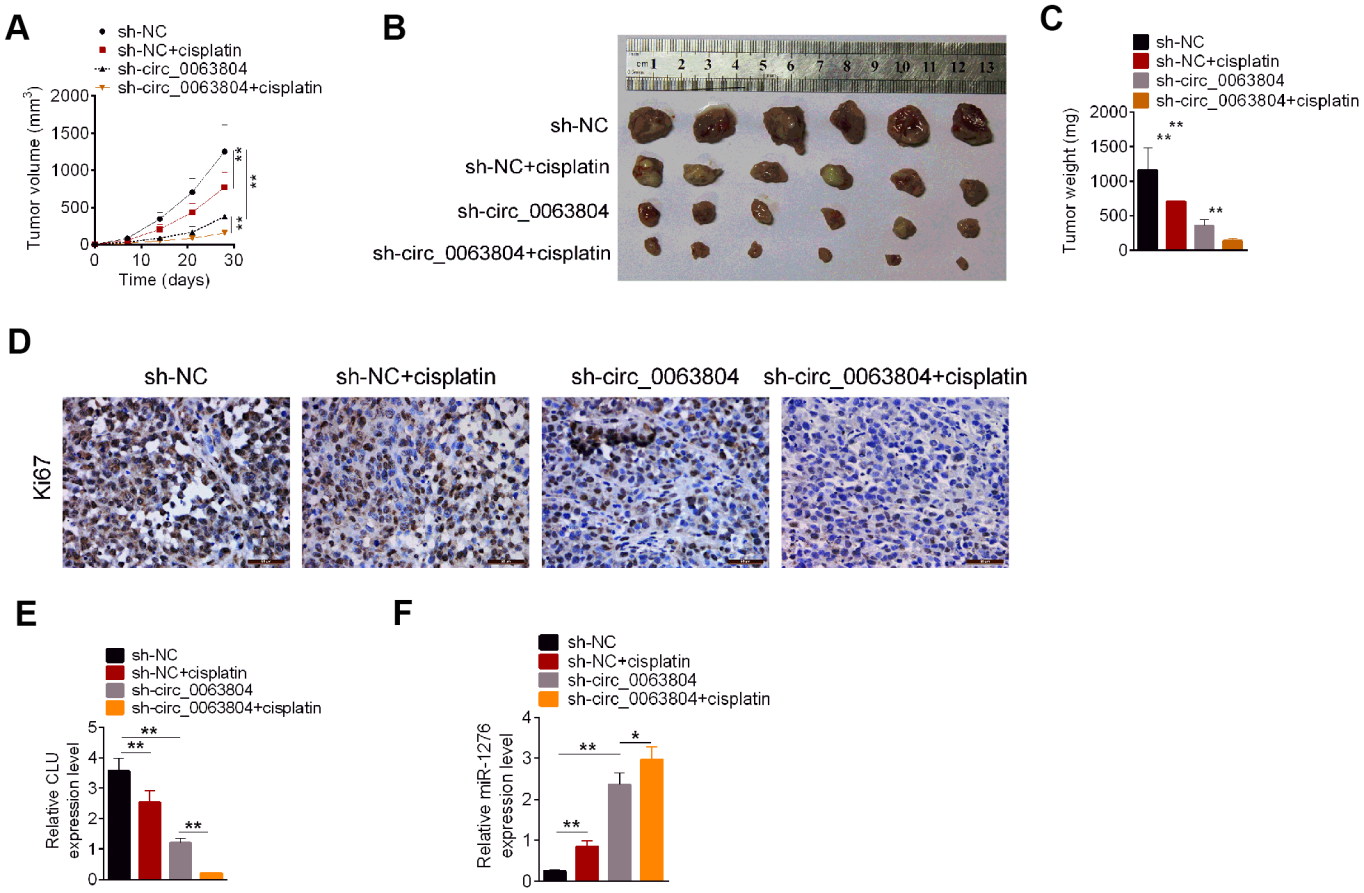
**Circ_0063804 silencing inhibited OC cells growth and resistance to cisplatin *in vivo*.** (**A**) Circ_0063804 silencing and cisplatin decreased xenograft tumor volume. (**B**) The xenograft tumor in nude mice of each group was presented. (**C**) Circ_0063804 silencing and cisplatin decreased xenograft tumor weight. (**D**) Circ_0063804 silencing and cisplatin reduced Ki76 expression in xenograft tumor. (**E**) Circ_0063804 silencing and cisplatin reduced CLU mRNA expression in xenograft tumor. (**F**) Circ_0063804 silencing and cisplatin increased miR-1276 expression in xenograft tumor. * *P* < 0.05. ** *P* < 0.01. OC: ovarian cancer.

The tumorigenicity and sensitivity to cisplatin of OVCAR3 cells was investigated. From Figure 6A–6C, seriously lower volume and weight of xenograft tumor was found in sh-NC + cisplatin group and sh-circ_0063804 group when matched with sh-NC group (*P* < 0.01). Matched with sh-circ_0063804 group, prominently lower volume and weight of xenograft tumor was observed in sh-circ_0063804 + cisplatin group (*P* < 0.01). The results of immunohistochemical tests of xenograft tumors showed that, the Ki76 positive cells in sh-NC + cisplatin group and sh-circ_0063804 group was less than sh-NC group. Meanwhile, xenograft tumors of sh-circ_0063804 + cisplatin group presented less Ki76 positive cells than that of sh-circ_0063804 group (Figure 6D). This study further assessed the expression of CLU mRNA and miR-1276 in xenograft tumor using qRT-PCR. Matched with sh-NC group, pronounced reduced CLU mRNA expression and elevated miR-1276 expression was found in xenograft tumor of sh-NC + cisplatin group and sh-circ_0063804 group (*P* < 0.01). Matched with sh-circ_0063804 group, prominently lower CLU mRNA expression and higher miR-1276 expression was displayed in xenograft tumor of sh-circ_0063804 + cisplatin group (*P* < 0.05 or *P* < 0.01) (Figure 6E, 6F).

## DISCUSSION

CircRNAs is non-coding RNAs with stable and conserved circular closed loop structure. It is capable of interacting with RNA-binding proteins, particularly miRNAs sponges [8]. CircRNAs is resistant to exonuclease. Thus it is used for diagnostic and prognostic biomarkers and therapeutic targets [9, 10]. At present, the function of some circRNAs in OC has been elucidated. For example, previous research had found that circ-SMAD7 was aberrantly up-regulated in OC. The reduced proliferation ability of OC cells was observed after circ-SMAD7 being silenced. Mechanically, circ-SMAD7 elevated OC cells metastasis and proliferation *in vitro* via inhibiting KLF6 expression [11]. The resistance to paclitaxel was one of the key reasons for the poor outcome of OC patients. Zhang et al. [12]. discovered that the overexpressed circCELSR1 in OC was correlated with paclitaxel resistance. circCELSR1 facilitated OC cells resistance to paclitaxel by enhancing FOXR2 expression via sponging miR-1252. In addition, circ_0061140, circ-CSPP1 and VPS13C-has-circ-001567 were reported to enhance proliferation and attenuate apoptosis of OC cells via sponging miRNAs [13–15]. Conversely, circ-ITCH, circ-ITCH, circ-0007874 and circ-LARP4 were served as tumor suppressor in OC, which inhibited OC cells growth by acting as competing endogenous RNAs to sponge miRNAs [16–19]. However, a large amount of novel circRNAs still needs to be deciphered. In this research, we noticed that high circ_0063804 expression indicated poor outcome of OC patients. circ_0063804 promoted malignant phenotype of OC cells, including enhancing growth and attenuating apoptosis. Moreover, circ_0063804 silencing declined the IC50 of OC cells, and associated with the down-regulation of p-gp and PARP protein and up-regulation of c-PARP. As reported in previous studies, p-gp and PARP protein was conductive to enhance cisplatin resistance, whereas c-PARP had the function of inhibiting cisplatin resistance [20–23]. Thi-Kim et al. [24] reported that the cleavage of PARP to c-PARP is a marker of apoptosis. The enhanced PARP cleavage is one of the reasons leading to the apoptosis of triple negative breast cancer cells. In this study, the silencing of circ_0063804 might promote OC cells apoptosis by promoting the cleavage of PARP. P-gp, coded by ABCB1, belongs to the ATP-binding cassette (ABC) membrane transport superfamily [25]. It has been found that p-gp was overexpressed in several human tumors, such as breast cancer and neuroblastoma [26, 27]. He et al. [28] reported that osteosarcoma cells with higher p-gp protein expression exhibited more resistant to cisplatin. The inhibition of p-gp increased the sensitivity of osteosarcoma cells to cisplatin. Thus they declared that p-gp could accelerate drug excretion and weaken chemotherapy efficacy. This research indicated that circ_0063804 silencing could reduce the expression of p-gp protein in OC cells. Therefore, circ_0063804 silencing might inhibit cisplatin resistance of OC cells by suppressing p-gp expression.

In this research, miR-1276 was confirmed as a target gene of circ_0063804. It was sponged by circ_0063804 in OC. Rescue experiment illustrated that miR-1276 inhibitor partially reversed the inhibition of circ_0063804 silencing on OC cells malignant phenotype and resistance to cisplatin. In previous study, miR-1276 was found to be sponged by lncRNA HCG11 and regarded as a cancer suppressor in gastric cancer [29]. Moreover, miR-1276 was identified to be potential tumor suppressor in breast cancer [30]. On the opposite, miR-1276 was researched as an oncogene in laryngeal cancer [31]. Meanwhile, highly expressed miR-1276 was reported in hepatocellular carcinoma tissues than that in normal liver tissues [10]. Our result indicated that miR-1276 was lower expressed in OC patients and directly inhibited by circ_0063804. miR-1276 inhibition partially reversed the inhibition of circ_0063804 silencing on OC progression and cisplatin resistance.

The function of CLU in malignant tumors has been controversial in recent years. A retrospective study reported that, CLU could facilitate tumor cell lines *in vitro* tumorigenesis and resistance to chemotherapy drugs. However, in mouse models, CLU could restrict the development of neuroblastoma and prostate cancer [32]. Previous researches reported that CLU was crucial in the inhibition of cell death, the modulation of pro-survival signaling, and the enhancement of chemotherapy resistance of tumor cells [33, 34]. In recent years, the expression and function of CLU in OC has been gradually emerged. It was revealed that CLU expression in recurrent resistant OC tissues was much higher than that in primary tumor tissues. High CLU expression was obviously related to poor survival of OC patients [35]. CLU was defined as an oncogene in OC, because the up-regulation of CLU was discovered to exacerbate angiogenesis of OC [36]. Moreover, the up-regulated CLU in OC was obviously associated with advanced stage and short average survival time [37]. In terms of the relationship between CLU and cisplatin resistance, it has been found that CLU silencing could promote the anti-tumor activity of cisplatin in human non-small cell lung cancer xenografts [38]. Simultaneously, CLU exerted a protective role against various kinds of apoptotic stimuli. It enhanced cisplatin resistance of bladder cancer by inhibiting apoptosis [39]. In this paper, it was revealed that CLU expression was up-regulated in OC cases. CLU overexpression partially reversed the inhibition of circ_0063804 silencing on OC progression and cisplatin resistance. Regarding the mechanism, circ_0063804 promoted OC development and cisplatin resistance by enhancing CLU expression through acting as a sponge of miR-1276.

This study had limitation. The role of CLU in relation to treatment with cisplatin should be explored. However, because of the limitations of laboratory conditions, it is unable to conduct more experiments to research this issue at present. This issue will be explored in our future study.

This article initially reported the function of circ_0063804 in OCmalignant development. Results demonstrated that high circ_0063804 expression was associated with worse outcome of OC patients. According to *in vitro*, circ_0063804 exacerbated OC cells proliferation and cisplatin resistance. Simultaneously, circ_0063804 silencing declined OC cells growth as well as cisplatin resistance *in vivo*. Mechanically, circ_0063804 facilitated OC progression and cisplatin resistance by promoting CLU expression through sponging miR-1276. Therefore, circ_0063804 was an oncogene in OC, which was suggested to be a potential target for OC clinical therapy.
